# Deriving the ultrastructure of α-crustacyanin using lower-resolution structural and biophysical methods

**DOI:** 10.1107/S0909049510034977

**Published:** 2010-11-05

**Authors:** Natasha H. Rhys, Ming-Chuan Wang, Thomas A. Jowitt, John R. Helliwell, J. Günter Grossmann, Clair Baldock

**Affiliations:** a Faculty of Life Sciences, University of Manchester, Manchester M13 9PL, UK; b School of Chemistry, University of Manchester, Manchester M13 9PL, UK; c STFC Daresbury Laboratory, Warrington WA4 4AD, UK

**Keywords:** α-crustacyanin, EM, SAXS, crystal packing of 1gka, PISA, analytical ultracentrifugation

## Abstract

The low-resolution structure of α-crustacyanin has been determined to 30 Å resolution using negative-stain electron microscopy (EM) with single-particle averaging. The protein, which is an assembly of eight β-crustacyanin dimers, appears asymmetrical and rather open in layout. A model was built to the EM map using the X-ray crystallographic structure of β-crustacyanin guided by PISA interface analyses. The model has a theoretical sedimentation coefficient that matches well with the experimentally derived value from sedimentation velocity analytical ultracentrifugation. Additionally, the EM model has similarities to models calculated independently by rigid-body modelling to small-angle X-ray scattering (SAXS) data and extracted *in silico* from the β-crustacyanin crystal lattice. Theoretical X-ray scattering from each of these models is in reasonable agreement with the experimental SAXS data and together suggest an overall design for the α-crustacyanin assembly.

## Abstract 2

The structure of α-crustacyanin has been determined to 30 Å resolution using negative-stain electron microscopy (EM) single-particle averaging and modelling with the β-crustacyanin dimer from the crystal structure (Protein Data Bank code 1gka), guided by PISA protein subunit interface calculations for 1gka, and compared with the protein arrangements observed in the crystal lattice of 1gka. This α-crustacyanin EM model has been checked against SAXS experimental data, including comparison with rigid-body models calculated from the SAXS data, and finally with analytical ultracentrifugation measurements.

## 1. Introduction

α-Crustacyanin is the carotenoid–protein complex responsible for the blue–black colouration of lobster carapace. Crustacyanins are members of the lipocalin family of hydrophobic ligand-binding proteins (Britton *et al.*, 1982 ▶). The carotenoid partner is astaxanthin, a natural fat-soluble red pigment found principally in plants, algae and photosynthetic bacteria. As well as their principal role in photosynthetic processes, carotenoids provide bright colouration, serve as antioxidants, and can be a source for vitamin A activity. Astaxanthin belongs to a group of oxygenated derivatives of carotenoids known as xanthophylls. These are commonly found in lobster and other seafood. In the assembly of α-crustacyanin, two genetically distinct apocrustacyanins (Chayen *et al.*, 2000 ▶; Cianci *et al.*, 2001 ▶; Habash *et al.*, 2004 ▶) each bind an astaxanthin molecule and form a heterodimer (β-crustacyanin). The crystal structure of β-crustacyanin (A1A3 dimer) has been determined and revealed two astaxanthin molecules held in close proximity (Cianci *et al.*, 2002 ▶). Eight β-crustacyanin dimers assemble to form α-crustacyanin, a 320 kDa complex containing 16 astaxanthin molecules. Astaxanthin has a UV-Vis absorption spectrum peak at a wavelength of 472 nm but upon binding to crustacyanin undergoes a large shift towards longer wavelengths (bathochromic) giving a blue-coloured protein complex (Cianci *et al.*, 2002 ▶). The β-crustacyanin dimer has a peak wavelength of 580 nm; however, a further bathochromic peak shift to 632 nm occurs in α-crustacyanin (Britton *et al.*, 1982 ▶). The mechanism of the additional wavelength shift in α-crustacyanin and the function of the protein–carotenoid complex is not understood. Theoretical and computational chemistry studies using the β-crustacyanin coordinates have been extensive, with two predominant theories for the bathochromic shift: firstly the proposed role of a protonated histidine (His 90 and His 92 are indeed found close to the keto oxygen of one end ring of each astaxanthin; Durbeej & Eriksson, 2006 ▶, 2003 ▶, 2004 ▶; Fisher *et al.*, 2009 ▶), and secondly an exciton interaction between the two polyene chains in β-crustacyanin (van Wijk *et al.*, 2005 ▶). Identifying the arrangement of β-crustacyanin dimers with the α-crystacyanin complex is anticipated to resolve the uncertainty surrounding this wavelength shift phenomenon. Possible suggestions for the function of α-crustacyanin include camouflage to avoid prey or as a primitive photoreceptor. Mimicking biological colouration properties by non-protein-bound carotenoids is under investigation *via* a growing ensemble of carotenoid crystal structures and intermolecular crystal packing arrangements (reviewed by Helliwell, 2008 ▶). Two small-angle X-ray scattering (SAXS) models have been reported in the literature. The first, by Dellisanti and co-workers (Dellisanti *et al.*, 2003 ▶), reported a helical arrangement. Chayen *et al.* (2003 ▶), by contrast, reported a piano stool type arrangement based on a fourfold symmetry assumption as a potential interpretation of an early electron microscopy (EM) study (Zagalsky & Jones, 1982 ▶). To date we have not been able to obtain well diffracting crystals to produce an atomic-level crystal structure of α-crustacyanin; therefore lower-resolution techniques have been used to identify the overall assembly of α-crustacyanin.

## 2. Experimental methods and results

For the EM, SAXS and analytical ultracentrifugation α-crustacyanin was extracted and purified from lobsters, sacrificed immediately prior to the extraction procedure, following the previously published purification procedure (Zagalsky, 1985 ▶). The experimental SAXS data as well as the Protein Data Bank (PDB) files for α-crustacyanin derived from the rigid-body best fit using 1gka to the SAXS data and the best fit using 1gka to the EM envelope data have been deposited with the IUCr^1^. The EM envelope data can be obtained directly from the authors by direct enquiry.

### 2.1. SAXS

SAXS experiments were performed at station 2.1 of the Daresbury SRS (Towns-Andrew *et al.*, 1989 ▶; Grossmann, 2002 ▶) using a 200 mm × 200 mm position-sensitive multiwire proportional counter operated at 512 × 512 pixels. Scattering data from α-crustacyanin were collected in the momentum transfer, *q*, range 0.005–0.64 Å^−1^.

### 2.2. Electron microscopy

**Figure 1:**
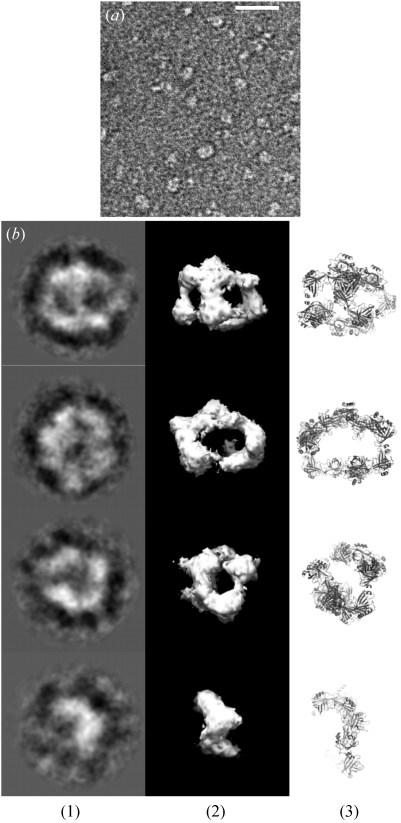
(*a*) One representative negatively stained EM image of α-crustacyanin from the 251 total images used to extract 10021 α-crustayanin particles. Scale bar = 50 nm. (*b*) Four representative class-averages (1), the EM three-dimensional reconstruction calculated by angular reconstitution (2), and views of the final docked model to the EM map (3). Box size = 33.6 × 33.6 nm. The final dimensions of α-crustacyanin are 130 Å by 140 Å by 180 Å. The analysis program used was *EMAN*.

Purified α-crustacyanin (6 µl) was allowed to absorb for 30 s onto a glow-discharged (30 s, 25 mA) carbon-coated 400 mesh copper grid. The grid was washed three times with water and then negatively stained with 4% (*w*/*v*) uranyl acetate pH 4.7. EM grids were observed using a FEI Tecnai Twin transmission electron microscope (TEM) equipped with a LaB_6_ filament operating at 120 keV. Images were recorded under low-dose conditions (Fig. 1*a* ▶) with −500 nm defocus at 52000× magnification on a 2048 × 2048 pixels CCD camera (TVIPS Tem Cam). The electron dose used for each image was typically 10 e^−^ Å^−2^. The final pixel size was 2.8 Å and images were converted to *IMAGIC5* format. Particles were selected automatically by the software *Boxer*, which is part of the *EMAN* package (Ludtke *et al.*, 1999 ▶). Three individual particles were selected from each micrograph for *Boxer* to base its selection criteria on. A total of 10021 particles were selected automatically. The selected particles were windowed into boxes of size 120 × 120 pixels. Band-pass filtering was used with a high-pass filter of 15 Å and a low-pass filter of 250 Å. The images were centred by cross-correlation to the total sum of the dataset. Class averages were calculated using reference free multivariate statistical analysis. A reference set of class averages was used to refine the alignment and for optimizing the class averaging. Angles were assigned to the best class averages and these were used to calculate a preliminary map. The three-dimensional reconstruction was refined iteratively until all images were incorporated. The Fourier shell correlation (FSC) was calculated in *EMAN* by comparing the models from odd- and even-numbered particles (van Heel & Schatz, 2005 ▶). The FSC at 0.5 indicated a resolution of 30 Å. The EM reconstruction showed an open assembly with no clear signs of symmetry.

### 2.3. PISA analyses

**Figure 2:**
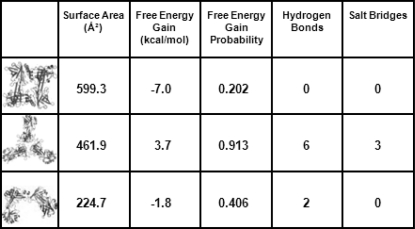
Thermodynamically stable interfaces predicted by PISA (Krissinel & Henrick, 2007 ▶). Favourable interfaces have multiple bonds, a large surface area and gain in magnitude of solvation free energy. The EM model is mostly composed of the lower two interface types.

The Protein Interfaces Surfaces and Assemblies (PISA) web server (http://www.ebi.ac.uk/msd-srv/prot_int/pistart.html) (Krissinel & Henrick, 2007 ▶) explores macromolecular interfaces and predicts probable quaternary arrangements or assemblies. Thermodynamically stable interfaces, based on the β-crustacyanin crystal structure 1gka (Cianci *et al.*, 2002 ▶), predicted by PISA are given in Fig. 2 ▶. A putative tetramer or ‘dimer of dimers’ has a surface area of 599.3 Å^2^ but has no salt bridges or hydrogen bonds identified. Another favourable quaternary arrangement has a trimeric structure with 461.9 Å^2^ surface area.

### 2.4. Protein docking to the EM map

β-Crustacyanin dimers (from 1gka) were docked into the EM envelope using the most energetically stable interfaces (Fig. 2 ▶) as determined by PISA. An interface is considered more probable if there is a large interface area, a high solvation free energy gain and multiple bonds present. The EM model for α-crustacyanin is asymmetrical and open, with evidence of internal holes (Fig. 1*b* ▶). The narrow density making up the structure suggests that the structure is made up of a chain of dimers.

### 2.5. Rigid-body modelling to the SAXS data

**Figure 3:**
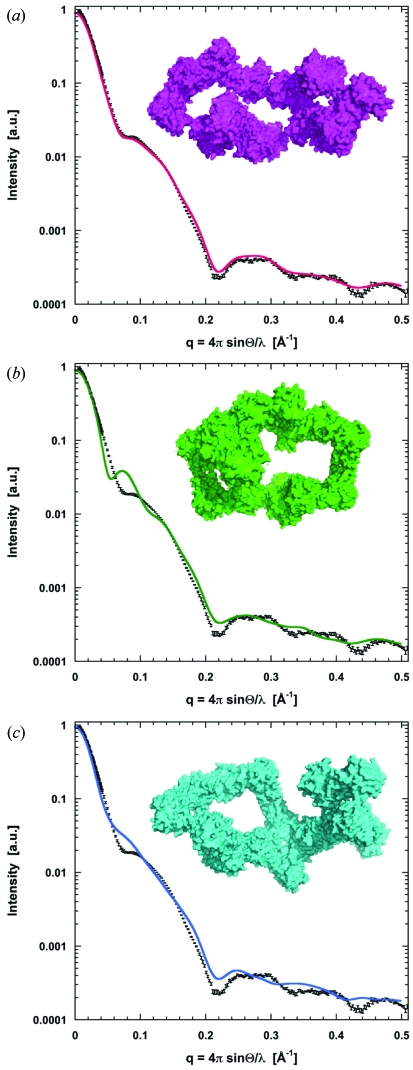
The experimental SAXS curve of α-crustacyanin with error bars (black) combining regimes of low and high scattering angles was collected at station 2.1 of the Daresbury SRS according to standard procedures (Grossmann, 2002 ▶) using sample-to-detector distances of 1 m and 4.5 m. SAXS curve of α-crustacyanin compared with the theoretical scattering of (*a*) the rigid-body model with lowest χ value (pink), (*b*) the EM-derived model (green) and (*c*) eight β-crustacyanin dimers extracted from the crystal lattice of 1gka (blue). In each case the model is shown as a surface representation.

Rigid-body modelling to the experimental scattering data was performed using *SASREF* (Petoukhov & Svergun, 2005 ▶). Eight β-crustacyanin dimers were fitted independently to the X-ray scattering data of α-crustacyanin. The simulations were repeated at least ten times with each set of parameters. The model with the lowest/best χ value represented an open elongated arrangement (Fig. 3*a* ▶).

### 2.6. Examination of 1gka crystal packing

The 1gka crystal packing layout has a very high solvent content of ∼85% (Cianci *et al.*, 2002 ▶). Visual examination of this layout reveals assembly possibilities that are also of an open layout. Two of these were extracted as PDB files and compared with experimental SAXS data of α-crustacyanin (§2.7).

### 2.7. Comparison of all theoretical models with the SAXS data

In order to compare the α-crustacyanin model built to the EM density map with experimental X-ray scattering data from α-crustacyanin, a theoretical SAXS curve for the model was calculated using *CRYSOL* (Svergun *et al.*, 1995 ▶). Fig. 3 ▶ shows a comparison of the theoretical and experimental scattering curves for α-crustacyanin. Although the χ value is relatively high, the curves show overall similarities across the scattering range suggesting the model is a good representation of the shape of α-crustacyanin. The model with the lowest χ value from rigid-body modelling was also compared with the experimental scattering data. The most plausible assembly of eight β-crustacyanin dimers extracted from the crystal lattice of 1gka was compared with the experimental X-ray scattering data.

### 2.8. Analytical ultracentrifugation (AUC)

**Figure 4:**
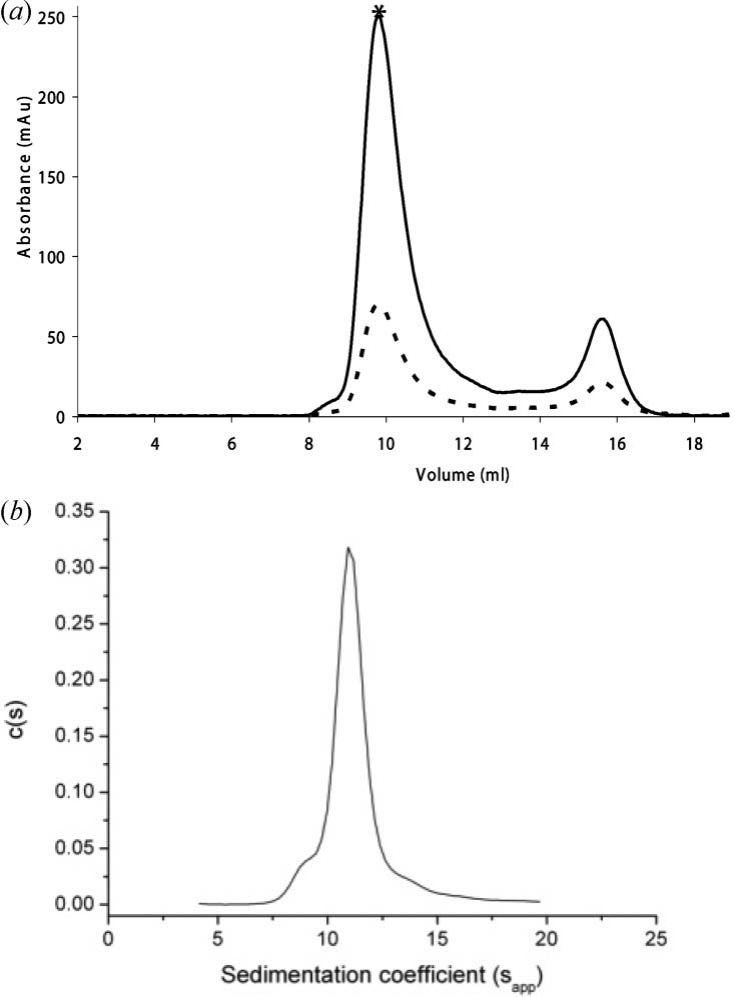
(*a*) Size exclusion chromatography profile of α-crustacyanin. The absorbance is measured at two wavelengths, 280 nm (dashed line) and 630 nm (solid line). The major peak corresponding to purified α-crustacyanin is indicated with an asterisk. There is a smaller peak eluting later which corresponds to β-crustacyanin. (*b*) *C*(*s*) analysis of α-crustacyanin derived from sedimentation velocity AUC. α-Crustacyanin has a sedimentation coefficient of 11.4 ± 0.5 S, hydrodynamic radius of 6.8 nm and frictional ratio of 1.5. The experimental sedimentation coefficient is very similar to the theoretical value of 11.1 S for the EM-derived α-crustacyanin model.

All experiments were performed in 10 m*M* sodium phosphate (pH 7.4) containing 0.15 *M* NaCl using a Beckman XL-A ultracentrifuge (Beckman Instruments, CA, USA) with an An50Ti-8-hole rotor fitted with the standard two-sector open-filled centrepiece for sedimentation velocity with quartz glass windows (Fig. 4 ▶). Velocity sedimentation analysis was performed at 40000 r.p.m. at 293 K, with the sedimenting boundary monitored every 90 s until the sample had fully sedimented. The protein concentration used was 0.2 mg ml^−1^. The data were interpreted with the model-based distribution of Lamm equation solutions *C*(*s*) using the software *Sedfit* (Schuck, 2000 ▶). The frictional ratio (*f*/*f*_o_) was calculated from the sedimentation coefficient. A bead model of the EM-derived α-crustacyanin model was generated with the solution modelling software *SOMO* (Rai *et al.*, 2005 ▶). The theoretical sedimentation coefficient of the model was 11.1 S which compares favourably with the experimental sedimentation coefficient of 11.4 ± 0.5 S for α-crustacyanin.

## 3. Discussion and conclusions

The negative-stain EM investigation to determine the α-crustacyanin nanostructure has yielded a new model (Fig. 1 ▶). Here we have shown at 30 Å resolution that the protein is asymmetrical and open. Predicted β-crustacyanin intermolecular interfaces have been incorporated and have helped eliminate bias when a subjective approach to the model building became necessary. The EM model has very similar theoretical hydrodynamic properties to the experimental sedimentation velocity AUC data. The hydrodynamic radius of α-crustacyanin was determined as 6.8 nm from the AUC data which is the same as the radius of gyration as measured by SAXS (Chayen *et al.*, 2003 ▶).

The theoretical scattering curve for the EM model is fairly consistent with the experimental SAXS curve to 30 Å resolution (*q* < 0.21 Å^−1^), which is the resolution limitation of this model. However, there is some deviation at ∼125 Å (*q* ≃ 0.05 Å^−1^) resolution which implies that there are some inconsistencies between the EM model and the actual structure of α-crustacyanin (χ = 10.4) (Fig. 3*b* ▶). The theoretical scattering curve for the SAXS rigid-body model with the lowest χ fitting value is also displayed in Fig. 3(*a*) ▶. This shows a good superimposition with the experimental data (χ = 5.8). The rigid-body model has an open elongated structure with loose packing between dimers. Additionally, a model of eight β-crustacyanin dimers extracted from the crystal lattice of 1gka was compared with the SAXS data (Fig. 3*c* ▶). The fit to the experimental data looks adequate with a slightly higher goodness-of-fit value (χ = 11.5) than that obtained for the EM model.

These three models for α-crustacyanin from EM, SAXS and the 1gka crystal lattice all have in common an open squashed-ring structure which would appear to have small interfaces stabilizing the assembly. The apparent high degree of hydration of α-crustacyanin would fit with the high solvent content in the β-crustacyanin crystal and the difficulties so far in growing diffraction quality crystals of α-crustacyanin. α-Crustacyanin has also been reported to be relatively unstable and can dissociate over time and in the presence of light to β-crustacyanin.

Structural models for α-crustacyanin proposed by the two reports published in 2003 (Chayen *et al.*, 2003 ▶; Dellisanti *et al.*, 2003 ▶) are at variance (even though the experimental SAXS profiles are essentially identical). Each report based their interpretation of the experimental data on a particular high-order symmetry (fourfold or helical symmetry). However, both symmetry constraints were put forward to be in harmony with the two models suggested from early EM data (Zagalsky & Jones, 1982 ▶) but lead to differing SAXS models. Detailed results for a possible fourfold symmetry model have not been pursued (published) until further more compelling EM data are available. The current results therefore provide important additional complementary insights and highlight that the overall shape is characterized by a less stringent stringing together of β-crustacyanin dimers as is also indicated by the observed crystal packing of β-crustacyanin (PDB code 1gka).

In order to improve the resolution of the EM reconstruction, further research should turn to using cryo-electron microscopy. For this method the sample can be visualized without unwanted effects from dehydration or negative staining. There is still the possibility that flattening of the sample upon adsorption to the EM grid has introduced some distortion. An alternative method is a renewed effort to produce new diffracting crystals of α-crustacyanin (Nneji & Chayen, 2004 ▶) along with microfocus X-ray beam scanning over the crystal, which, even if they only diffracted to 10 Å resolution, would be a significant further step forward in the pursuit of the structure of α-crustacyanin. Nevertheless, this 30 Å EM reconstruction and models presented herein provide a good basis for future work.

## Supplementary Material

Supplementary material file. DOI: 10.1107/S0909049510034977/ys5057sup1.txt

Protein Data Bank files for alpha-crustacyanin derived from the rigid-body best fit using 1gka to the SAXS data. DOI: 10.1107/S0909049510034977/ys5057sup2.pdb

Protein Data Bank files for alpha-crustacyanin derived from the rigid-body best fit using 1gka to the EM envelope data. DOI: 10.1107/S0909049510034977/ys5057sup3.pdb

## Footnotes

1 Supplementary data for this paper are available from the IUCr electronic archives (Reference: YS5057). Services for accessing these data are described at the back of the journal.
